# Highly Sensitive Plasmonic Waveguide Biosensor Based on Phase Singularity-Enhanced Goos–Hänchen Shift

**DOI:** 10.3390/bios12070457

**Published:** 2022-06-26

**Authors:** Manel Hedhly, Yuye Wang, Shuwen Zeng, Faouzi Ouerghi, Jun Zhou, Georges Humbert

**Affiliations:** 1 XLIM Research Institute, UMR 7252 CNRS/University of Limoges, 123 Avenue Albert Thomas, 87060 Limoges, France; manel23hedhli@gmail.com (M.H.); yuyewang@link.cuhk.edu.hk (Y.W.); georges.humbert@xlim.fr (G.H.); 2 Faculty of Sciences of Tunis, Université de Tunis El Manar, 2092-El Manar, Tunis 1068, Tunisia; faouzi23ouerghi@gmail.com; 3 Bionic Sensing and Intelligence Center, Institute of Biomedical and Health Engineering, Shenzhen Institutes of Advanced Technology, Chinese Academy of Sciences, Shenzhen 518055, China; 4 Department of Biomedical Engineering, The Chinese University of Hong Kong, Shatin, N.T., Hong Kong 999077, China; 5 Light, Nanomaterials & Nanotechnologies (L2n), CNRS-ERL 7004, Université de Technologie de Troyes, 10000 Troyes, France; 6 Department of Microelectronic Science and Engineering, School of Physical Science and Technology, Ningbo University, No. 818 Fenghua Road, Ningbo 315211, China; zhoujun@nbu.edu.cn

**Keywords:** surface plasmon resonance biosensors, optical sensor, plasmonic waveguide

## Abstract

The detection for small molecules with low concentrations is known to be challenging for current chemical and biological sensors. In this work, we designed a highly sensitive plasmonic biosensor based on the symmetric metal cladding plasmonic waveguide (SMCW) structure for the detection of biomolecules. By precisely designing the configuration and tuning the thickness of the guiding layer, ultra-high order modes can be excited, which generates a steep phase change and a large position shift from the Goos–Hänchen effect (with respect to refractive index changes). This position shift is related to the sharpness of the optical phase change from the reflected signal of the SPR sensing substrate and can be directly measured by a position sensor. Based on our knowledge, this is the first experimental study done using this configuration. Experimental results showed a lateral position signal change > 90 µm for glycerol with a sensitivity figure-of-merit of 2.33 × 10^4^ µm/RIU and more than 15 µm for 10^−4^ M biotin, which is a low molecular weight biomolecule (less than 400 Da) and difficult to be detected with traditional SPR sensing techniques. Through integrating the waveguide with a guiding layer, a strong improvement in the electric field, as well as sensitivity have been achieved. The lateral position shift has been further improved from 14.17 µm to 284 µm compared with conventional SPR substrate with 50 nm gold on single side. The as-reported sensing technique allows for the detection of ultra-small biological molecules and will play an important role in biomedical and clinical diagnostics.

## 1. Introduction

Biosensors are tools for analyzing biomaterials to understand their composition and functions. They are generally composed of: (i) a molecular recognition element, or a probe, whose role is to react with the target molecule to be detected (the analyte). (ii) A transducer, on which the ligand is fixed. It converts the interaction event between the ligand and the analyte into a physical signal that is more easily measured and interpreted. (iii) A signal processing system at the output of the transducer, allowing the acquisition and recording of the measurement. The last decades have proven that the integration of low-cost biosensors in a variety of fields has become indispensable [1,2,3,4]. They are generally capable of detecting various biological molecules [5,6,7,8], where molecular interactions can be monitored by optical or electronic signal change. Applications using biosensors include general health monitoring [9], critical disease review [10], disease analysis and diagnosis [11]. They also play an important role in veterinary and agricultural applications [12,13], food processing and monitoring [14], environmental pollution monitoring and other applications [15,16].

In particular, the detection of low molecular weight biomolecules at extremely low concentration levels has attracted much attention in these years. Some researchers have resorted to the technique of labeling small molecules with a fluorescent label [17,18] or a metallic nanoparticle [19,20] in order to facilitate detection, but this process remains invasive since it may alter the functionality of the molecule, especially when the size of the label is bigger than the targeted molecules. Therefore, label-free techniques are more flexible and highly demanded. Among these methods, the surface plasmon resonance (SPR) technique that offers the merits of label-free, real-time and low-cost sensing, has been exploited in numerous biosensing applications, such as exosome detection [21], nucleic acid detection [22], immunoglobulin G (IgG) detection [23], etc. [24]. SPR is a physical phenomenon of the light–matter interaction, mainly known for its strong ability to enhance the electromagnetic field through an electron wave on the surface of a metallic structure. However, it is still quite challenging for the detection of small target analytes of low molecular weight, especially for cancer biomarkers (e.g., TNF-α and I1L-α), thyroid hormones and bacterial pathogens in infectious diseases [25,26]. Moreover, the detection of biological and chemical molecules of concentration less than 1 fM (10^−15^ mol/L) in complex matrices, such as urine, saliva and blood serum is also a challenge. Recently, scientific studies have proven that both challenges can be overcome based on the optical phase singularity interrogation method [27,28]. At the resonance angle, a total energy transfer from the incident light to the SPR will occur at the sensing interface [29,30,31], which leads to a complete decrease in reflectivity and thus an extreme phase singularity. This extreme singularity in the phase signal is highly sensitive to the refractive index change induced by the molecular binding at the plasmonic sensing substrate.

Further, a higher order of the phase signal, the Goos–Hänchen (GH) shift, will lead to an improvement in sensitivity. The GH shift effect refers to the phenomenon that when a light beam is totally reflected at the boundary between different media, it will shift from the position predicted by the geometrical optics. This phenomenon is explained by the fact that the incident light penetrates the second medium and creates an evanescent wave that will propagate along the interface over a certain distance before re-emerging in the first medium. This phenomenon was first demonstrated experimentally by Goos and Hänchen in 1947 [32,33]. This large Goos–Hänchen (GH) position shift has attracted the interest of several researchers for its potential applications in integrated optics, optical storage and optical sensors [34]. Various theoretical [35,36,37,38] and experimental [39,40] studies have been proposed. Furthermore, the GH shift is very sensitive to any modification of the materials and the structures leading to a sharp variation of the optical phase signal, which makes this SPR sensing method more sensitive than the ones based on angle shift measurements. For example, the studies conducted by Yin et al. [39] showed a significant shift for the case of a Kretschmann configuration. A plasmonic metasensor formed by atomically thin perovskite nanomaterials has also been proposed for improving the GH sensitivity up to 1.5458 × 10^9^ µm/RIU [41], offering numerous opportunities for the detection of chemical and biological markers difficult to identify (e.g., the diagnosis of tumor necrosis factor—TNF). We can also mention the theoretical studies of GH shift, developed on a symmetric graphene cladding waveguide, showing a sensitivity up to 2.2 × 10^5^ μm/C, which could open avenues for applications of optical temperature sensors [42].

Among a variety of techniques used to excite the SPR, the most commonly applied ones are based on prism or grating coupling structures [41,43]. The sensitivity of grating coupling structures is lower than that of the prism coupling structures [44]. Moreover, its manufacture is more expensive due to the additional complexity of the gratings [45]. In contrast, prism-based SPR biosensors offer more advantages, such as low manufacturing cost, simpler operating flexibility and high sensitivity. Nevertheless, the applicability of the structures is limited in some cases since the refractive index (RI) of the analyte must be lower than that of the prism [46]. In addition, several configurations have also been proposed based on plasmonic-based optical fibers, including tapered fibers, D-type fibers, single mode fibers (SMFs), multi-mode Fibers (MMFs), Bragg-grating fibers and Wagol wheel fibers [47,48,49]. These fiber-based plasmonic sensors can direct the incident light at a narrow angle and provide a wide operating range. However, it is still very challenging to reach the detection limit of 10^−8^ RIU. In order to overcome these drawbacks, symmetric metal cladding waveguide (SMCW) structures have been investigated [50,51]. These studies have shown that the sensitivity is strongly improved using this structure compared to the single layer of gold substrate coupled with prism. The higher sensitivity is resulted from the ultra-high order excited mode created in the guiding layer, which greatly shortens the time and distance of the light–sample interaction. Since the sample is directly injected into the guiding layer, it is placed in the oscillating field region where the interaction strength with the sample is stronger than the evanescent field excited with a single layer of gold substrate coupled with prism.

Here, we report the first biosensing demonstrations based on GH shift measurement of a prism-associated SMCW, achieving ultra-high sensitivity with a detection limit down to 10^−12^ refractive index units (RIU). Our SMCW allows for a strong electric field enhancement, resulting in a significant increase in the GH shift from 18 µm to 284 µm compared to the four-layer output of the nanophotonic cavity at the same wavelength [52]. Moreover, this value is about 20 times higher than that associated with the case of using an Au substrate only. Furthermore, we have experimentally demonstrated the efficiency and accuracy of this structure in detecting extremely small refractive index (RI) changes, which is of great importance for real-time detection applications. This performance has been exploited to detect small molecules, such as biotin (MW = 244.31 Da), at low concentrations, which remains a challenge for traditional biosensors. Our proposed configuration proved its ability to detect ultra-small refractive index (RI) changes (10^−8^ RIU), which is an ultra-low value compared with the results found with the multiplexed label-free imaging sensor [53] and those with the common-path interferometric sensor, which are in the detection limit level of 10^−6^ RIU [54]. We also recorded a sensitivity figure of merit equal to 2.33 × 10^4^ µm/RIU, which is more than three orders of magnitude higher than sub-wavelength phase-shifted Bragg grating waveguide [55], subwavelength grating metamaterial (SGM) waveguide [56] and automatic portable biosensing system based on the microring resonator [57].

## 2. Design and Principles

**Figure 1:**
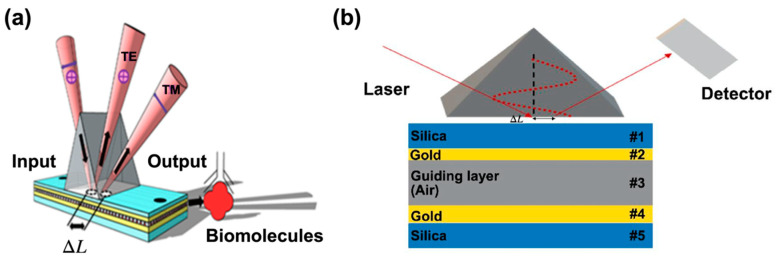
(**a**) Schematic of the symmetrical metal-cladding waveguide biosensor; (**b**) principle model of coupling in SMCW.

The schematic of the SMCW biosensor structure is shown in Figure 1a. It consists of an equilateral prism made of SF11 glass in contact with a SMCW (through an optical matching oil). The plasmonic waveguide (SMCW) is composed of five layers, in which there is a 45 nm thick silica layer and a 35 nm thick gold layer serving as a coupling layer, a 1.1 mm thick air gap (sensing area) and a 110 nm thick gold layer. The air gap layer serves as a guiding layer by multiple reflections of the light beam on both gold layers leading to the excitation of ultra-high order modes inside the guide [58].

Here, Figure 1a TE refers to the transverse electric waves, also sometimes called H waves, characterized by the fact that the electric vector (E) is always perpendicular to the direction of propagation. TM represents the transverse magnetic waves, also called E waves, characterized by the fact that the magnetic vector (H vector) is always perpendicular to the direction of propagation, respectively. ΔL is the lateral position shift.

The advantage of using the SMCW waveguide is its strong ability to trap light in the guiding layer where the analyte will be injected, which consequently causes a strong absorption in the visible and near infrared range.

When the light beam is incident from the air and transmitted through the SF11 glass prism, the silica layer (#1) and reaches the metal surface (#2), it generates an evanescent field on the surface of the metal layer. Since the thickness of the waveguide layers were fine-tuned to have a large lateral phase change, as well as a positional change, the thickness of the first metal layer (coupling layer) was about several tens of nanometers, which helps the evanescent field to reach the interface of the guided wave layer of the metal (#4). This will create an opposite evanescent field on the detection interface (#3), as shown in Figure 1b. Due to the interaction of these two oppositely directed fields, the incident light can be coupled into a waveguide.

Under resonant conditions, when the light beam reaches the gold layer, a large part of the light energy will be transferred into the guiding layer. The SPR resonance will lead to the attenuated total reflection (ATR) spectrum. This spectrum describes the relationship between the reflectivity and the incident angle or wavelength of the reflected light. The reflection coefficient of the optical system for the TE mode is written as follows [59]:

(1) $$r=\frac{r_{32}-r_{32}r_{21}^{2}\exp\left(2ik_{1}d\right)+r_{21}\exp\left(2ik_{2}h\right)-r_{21}\exp\left(2ik_{1}d\right)\exp\left(2ik_{2}h\right)}{1-r_{21}^{2}\exp\left(2ik_{1}d\right)+r_{32}r_{21}\exp\left(2ik_{2}h\right)-r_{32}r_{21}\exp\left(2ik_{1}d\right)\exp\left(2ik_{2}h\right)}$$

Here, $r_{ij}=\frac{\left(k_{i}-k_{j}\right)}{\left(k_{i}+k_{j}\right)}$, which represents the complex Fresnel reflection coefficient for the boundary between media *i* and *j*. The normal components of the wave vector in layer *j* ($k_{j}$) and the propagation constant of guided modes in layer 3 (β) are expressed as:

(2) $$\;\;\;\;k_{j}=\sqrt{\left(k_{0}^{2}\varepsilon_{j}-\beta^{2}\right)};\;(j=1,\;2,\;3)$$

(3) $$\beta =k_{0\;}\sqrt{\varepsilon_{3}}\;\sin\theta$$

*h* and *d* are the thicknesses of gold layer (#2) and the guided layer (#3), respectively. $k_{0\;}$ = $2\pi /\lambda_{0}$ is the wave vector of the optical wave in free space, and $\varepsilon_{j}$ is the dielectric constant for the *i*-th layer. *θ* and $\lambda_{0}$ are the incident angle and wavelength, respectively.

To evaluate the detection performance of our SMCW biosensor, we chose a Kretschmann excitation method, as shown in Figure 1. The thickness of the coupling layer has been well optimized to achieve a condition very close to zero reflection. When the incident light beam is totally reflected on a dielectric interface, a lateral shift from the position predicted by the optics occurs; this phenomenon is called the GH effect, which has been cited in the introduction.

It is known that the SPR reflectivity is changed with a function of the incident angle under the excitation of a guided mode. When matching the condition of resonance, the reflectivity decreases considerably. This will significantly increase the reflective GH shift and forms a peak, which corresponds to the minimum of reflectivity. The enhancement of the GH effect in the ultra-high order mode in SMCW can be explained in the context of energy flow conversion [60]. Indeed, when the incident light arrives on the first gold layer the energy is divided into two parts: one part will be directly reflected and another part will be guided laterally in the form of an oscillating wave field. By propagating laterally along the SMCW, this field continues to interact with the incident medium. Finally, the two wave fields (reflected mode and leaked mode) are superimposed giving a giant reflected light that is laterally shifted from the position of the predicted light. Under this near-zero reflection phenomenon, the rapid drop in reflected light intensity is accompanied by an extremely abrupt phase change at the resonance angle, which can be exploited to significantly improve the sensitivity of plasmonic sensors.

To calculate the GH shift, we used the transfer matrix method (TMM) with Fresnel equations. We considered p-polarized light because it undergoes an abrupt phase change as long as the plasmon resonance is excited compared to no phase change for the s-polarized light. Thus, the transfer matrix for a multilayer model is described as [41]:

(4) $$M=\left[\begin{array}{cc} M_{11} & M_{12}\\ M_{21} & M_{22} \end{array}\right]=\overset{N-1}{\underset{m=2}{\prod }}\left[\begin{array}{cc} \cos\beta_{m} & -\frac{i(\sin\beta_{m})}{q_{m}}\\ -i\left(\sin\beta_{m}\right).q_{m} & \cos\beta_{m} \end{array}\right]$$

(5) $$q_{m}=\frac{\sqrt{\varepsilon_{m\;}-n_{1\;}^{2}\sin^{2}\theta_{inc}}}{\varepsilon_{m\;}}$$

(6) $$\;\beta_{m}=\frac{2\pi d_{m}}{\lambda_{0}}\sqrt{\varepsilon_{m\;}-n_{1\;}^{2}\sin^{2}\theta_{inc}}$$

In addition, $\;\theta_{inc}$ in Equations (2) and (3) is the angle of incidence, we define the Fresnel reflection coefficient for p-polarized light as following:

(7) $$\;r_{p}=\frac{(M_{11}+M_{12}q_{N})q_{1}-\left(M_{21}+M_{22}q_{N}\right)}{(M_{11}+M_{12}q_{N})q_{1}+\left(M_{21}+M_{22}q_{N}\right)}$$

At the dark point, when the value of the reflected light intensity is close to zero, a large phase shift ($\Phi_{p}$) is observed. This phase shift causes a large lateral shift GH (*L_shift_*) defined as follows [61]:

(8) $${\;\Phi }_{p}=\arg\left(r_{p}\right)$$

(9) $$L_{shift}=-\frac{\lambda_{inc}}{2\pi \sqrt{\varepsilon_{1}}}\;\frac{\Delta {\;\Phi }_{p}}{\Delta \theta_{inc}}$$

We defined the SPR sensitivity based on Goos–Hänchen (GH) shift as:

(10) $$\;S_{GH}=\frac{\Delta L_{shift}}{\Delta n_{target}}$$

**Figure 2:**
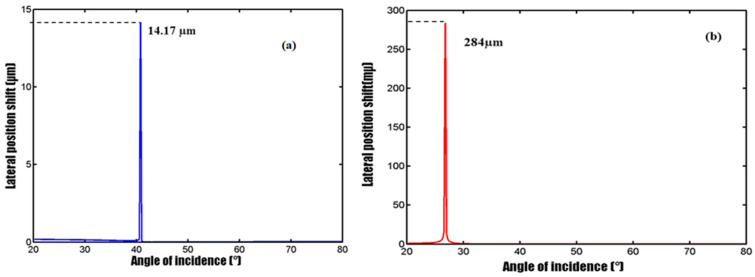
Simulation results of GH lateral position shift (**a**) with a gold substrate, (**b**) with the SMCW structure.

The lateral position change of the Au-only substrate and our SMCW structure was simulated. Figure 2 clearly shows that with our optimized waveguide configuration, an extremely giant lateral position shift was obtained, which reaches up to 284 μm at the resonance angle. This value is about 20 times larger than that associated with the case of using the Au-only substrate, demonstrating the sensitivity enhancement offered by the SMCW structure.

## 3. Experiments

### 3.1. Device Fabrication

**Figure 3:**
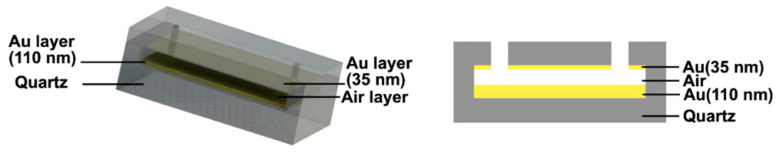
Symmetrical metal-cladding plasmonic waveguide dimensions.

The SMCW was fabricated with the electron beam evaporation method to deposit gold films on two pieces of quartz glass sheets (Figure 3). The quartz glass sheet of the upper layer of the waveguide is composed of liquid inlet/outlet holes and a deposited gold film thickness of 35 nm. The quartz glass sheet of the lower layer was coated with a 110 nm thick gold film. The two quartz glass sheets were bonded with an optical glue to construct an air gap layer of 1.1 mm thickness (i.e., the hollow-core of symmetrical metal-cladding waveguide). When preparing the symmetrical metal-cladding waveguide, all components are optically contacted with excellent parallelism.

### 3.2. Experimental Setup

**Figure 4:**
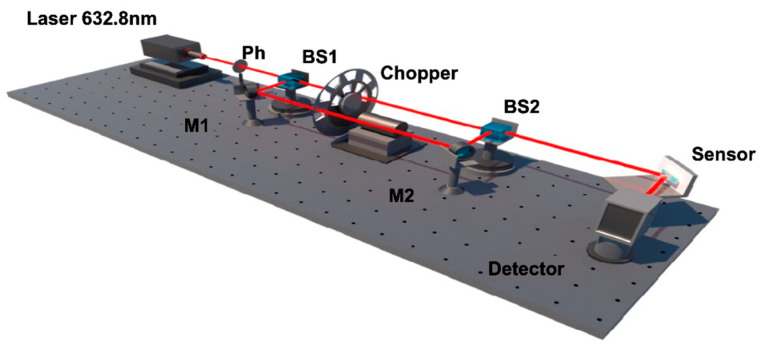
Experimental setup for measuring the differential lateral position shift (*L_shift_*).

The experimental configuration is shown in Figure 4. A He–Ne laser is used as an excitation light source. A pinhole is placed directly at the output of the laser to filter stray light. The light beam is split into p-polarized and s-polarized light beams via a polarized beam splitter. An optical chopper is used to alternate *p* and *s* polarization excitation on our SMCW biosensor.

The injections of the liquids (biomolecules, analytes…) into the sensing area are done with a syringe pump through the inlet and outlet tubes embedded in the upper quartz glass sheet. A lateral position detection detector is used to record in real time the position of the reflected light beam for both (*p* and *s*) polarizations. This signal is collected via a data acquisition board and analyzed using LABVIEW and MATLAB programs. When the liquid samples were injected into the guiding layer, a real-time measurement of the phase singularity-related lateral position shift was performed. These lateral shifts are explained by the binding produced by the target molecules to the sensing surface.

### 3.3. Chemicals

In our measurements, PBS (phosphate buffered saline) solution with different concentrations (25%, 50%, 75% and 100%) was used. This solution contains sodium chloride, disodium phosphate, monopotassium phosphate and potassium chloride. It is mainly used to rinse the cell (sensing area) when it is necessary to remove all traces of medium before processing them [52]. Before any injections of the biomolecules, a diluted solution of (3-Aminopropyl) trimethoxysilane (APTMS) was injected with the aim of attaching the molecules to the substrate by creating a covalent bond between the mineral and organic components. Biosensing measurements were realized for different concentration of glycerol solutions, bovine serum albumin (BSA) solutions and biotin solutions. All chemicals were purchased from Sigma Aldrich, Saint. Quentin Fallavier, France.

## 4. Results and Discussion

### 4.1. Experimental and Theoretical Results

**Figure 5:**
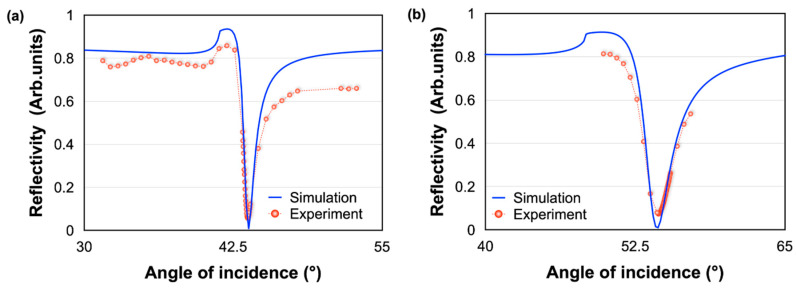
Reflectivity spectra measurement with prism–Au substrate with (**a**) air and (**b**) water in the sensing cell.

The difference of the properties between a gold substrate and the SMCW are investigated by measuring the angular scanning reflectivity spectra (ATR spectrum) with air and then water as sensing medium. As expected, a clear drop of reflected light beam is observed (at 43 degrees) when the angle of incidence of the light beam matches the excitation conditions of the plasmonic wave through the prism (Figure 5a). This angle shifts approximately 10 degrees when water is injected in the sensing area (Figure 5b). It is worth noting the good agreement between theoretical and experimental results, which subsequently confirms the reliability of our configuration and serves as a good calibration for the evaluation of the detection performances. This step is used to fix the dark angle (the angle that is the minimum intensity of reflection) of the prism that will be used for the prism waveguide measurements.

The ATR spectrum of the SMCW structure is measured by referencing the angle of incidence to the one of the gold substrates. The waveguide was fixed on a rotating stage, aligned with the laser and the pinhole, which served to make an angular scan. As the rotating stage rotates, the angle of incidence changes, while the high precision optical power meter (Newport 2832C) continues to measure the intensity of the reflected light continuously. We made measurements for −3.5 degrees of angular rotation around the dark angle for air and then for water.

**Figure 6:**
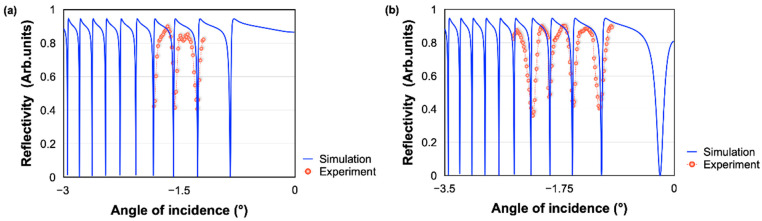
Reflectivity spectra measurement with the SMCW waveguide with (**a**) air and (**b**) water in the sensing cell.

As shown in Figure 6, when the incident light energy is coupled into the guided modes, the reflected light intensity R = |r| 2 decreases significantly and a series of reflection dips in the ATR spectrum are produced in contrast to the results found previously with the gold substrate alone. The simulation of the ATR spectrum with a MATLAB program based on the Fresnel equation is shown in Figure 6. We have also experimentally measured the reflectivity under different incident angles. The experimental and theoretical results show a well-matched trend. The measured reflectivity values in some points are deviated from the theoretical results, which may be caused by the surface roughness of the sensing substrate. By analyzing the two curves (a and b), it can be seen that the reflection troughs become increasingly tight as we approach the dark angle for water, which proves that the effective refractive index of the ultra-high order modes is directly related to the refractive index of the analyte. These attractive properties confirm the relevance of the SMCW waveguide structure for sensing biomolecules. It is worth noting that in addition to offering a higher sensitivity, GH shift measurement is also an easier and faster method than the ones based on measuring variations in these complex ATR spectra.

### 4.2. Biosensing Results

After having well positioned the SMCW with the prism in the GH shift measurement setup (i.e., position of the dark angle), a series of injection of glycerin solutions with different concentrations was realized to evaluate its sensitivity. Glycerin, also known as glycerol, is a fat that occurs naturally in skin, vegetable oils and animal fats. It is generally presented in the form of a rather viscous and thick liquid, colorless and odorless and it is miscible with water.

**Figure 7:**
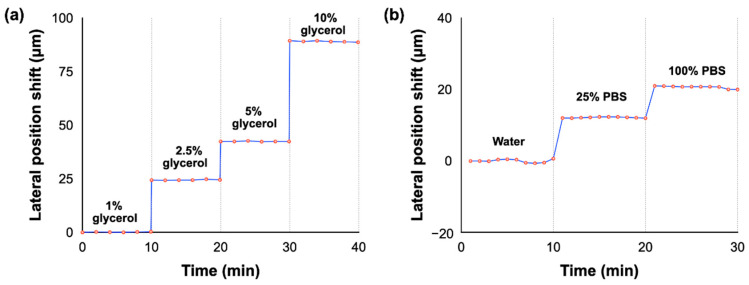
Real-time differential lateral position shift measurement for various weight ratios of (**a**) glycerin and (**b**) PBS solutions.

Different weight ratios (1%, 2.5%, 5% and 10%) of glycerin solutions were injected into the waveguide chamber, while the GH shift is measured in real time (Figure 7). These solutions lead to a refractive index variation from 1.333 to 1.46716 (from water to 10% glycerin). As expected, the lateral position shift has a linear relationship with the glycerol/water weight ratios. Following the injection of 1% glycerol (0.0012 refractive index unit, RIU) into the waveguide chamber, the lateral shift reaches 28 µm, which corresponds to a figure of merit of sensitivity about 2.33 × 10^4^ µm/RIU (after the saturation time ~10 min). The lateral shift evolution keeps the same pattern for all glycerol concentrations after reaching the saturation time.

In addition to that, as a standard sensor evaluation procedure, we also sequentially injected PBS solutions of different concentrations into the guide layer. The lateral positional shifts of water, of 25% and 100% concentration PBS solutions, are plotted in Figure 6b. The water solution is taken as reference. The lateral shift from the water solution is about 20 µm for the 100% PBS solution. These measures clearly emphasis the strong influence of the refractive index variation (in the chamber) on the GH shift (lateral shift position) demonstrating the high relevance of our SMCW for sensing biomolecules.

**Figure 8:**
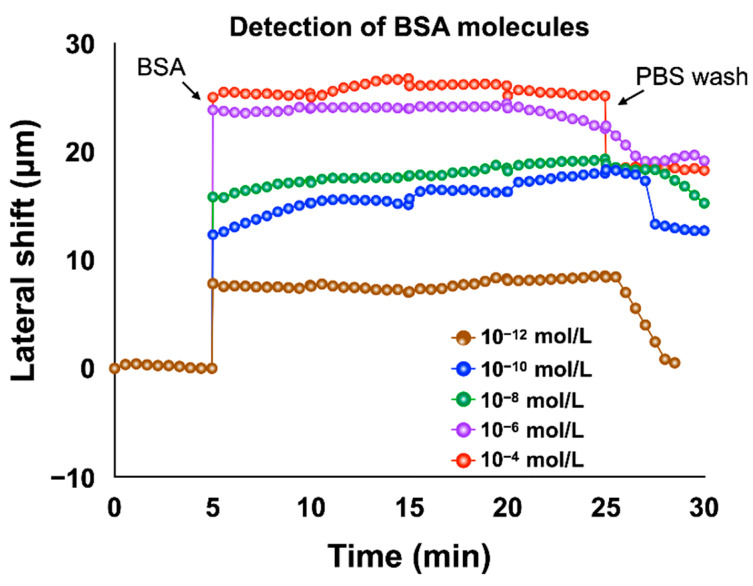
Real-time detection of different concentration of BSA solutions based on lateral position shift measurement.

The bovine serum albumin (BSA) molecule is a protein extracted from bovine serum. It is widely used in various bioassays such as ELISA (Enzyme-Linked Immunosorbent Assay), immunohistochemistry and Western blot. This molecule has a relatively high molecular weight (66,463 Da). In this section, we present the results of measurements of BSA solutions of different concentrations. Before injecting our solutions, we first injected a chemical linker (3-Aminopropyl) trimethoxysilane (APTMS) in order to create an effective binding to the substrate and after each detection analysis; we injected PBS to remove the excess of molecules not bound to the substrate. Following this injection, the lateral shift barely changed as most of the molecules were bound to the substrate. The evolution of the lateral position shift is mainly explained by the binding produced between the biomolecules and the substrate. We measured different solutions with concentrations ranging from 10^−12^ M to 10^−4^ M. Upon injection of the 10^−12^ M concentration BSA solution, the lateral position shift shows an abrupt change immediately and stabilizes around 8.5 µm, as shown in Figure 8. For the 10^−10^ M concentration solution, the lateral position shift reaches 12.5 µm after the saturation time (20 min). The lateral shift changes for BSA solutions with 10^−8^ M, 10^−6^ M and 10^−4^ M concentrations all had similar trends and stabilized at around 18.9 µm, 24.2 µm and 26.5 µm respectively after the saturation time. Decreasing the concentration of BSA solutions causes a linear decrease in the lateral shift, as shown in Figure 8.

**Figure 9:**
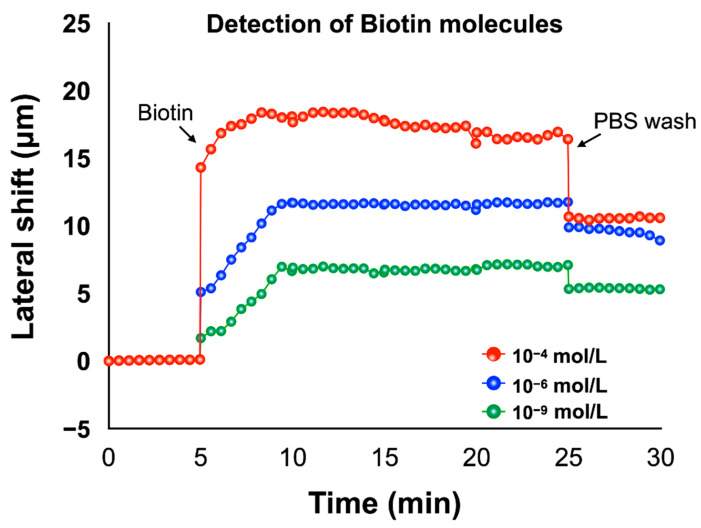
Real-time detection of biotin molecules based on lateral position shift measurement.

In addition to BSA molecules, we also detected biotin molecules that have a very low molecular weight, around 244 Da, which makes it difficult to be detected. Recently, results from nanomaterials enhanced plasmonic sensors can be used to improve the detection limit from nanomolar to picomolar [62]. However, these structures are complicated and expensive. Here, we present the results of measurements of biotin solutions of different concentrations using our low-cost SMCW biosensor. Similar to BSA detection, we first injected the chemical linker before biotin detection and used buffer solutions to remove the excess biomolecules. In Figure 9, it can be seen that the lateral shift changes progressively with increasing concentration and saturates at about 7 µm for a biotin concentration of 10^−9^ M. Thus, for a biotin solution of 10^−6^ M, the lateral evolution of the position shift as a function of concentration follows the same pattern and stabilizes around 12 µm after the same saturation time (20 min). Finally, for 10^−4^ M biotin concentration, the evolution of the lateral shift as a function of concentration reaches 18 µm after 20 min.

**Figure 10:**
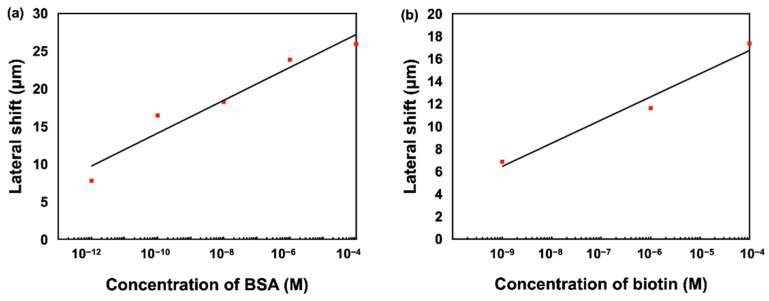
Lateral position shift measurement of (**a**) BSA (**b**) biotin molecules with different concentration levels.

The differential lateral position shift signals acquired under concentrations of BSA ranging from 10^−12^ to 10^−4^ M, and biotin ranging from 10^−9^ to 10^−4^ M were also summarized in Figure 10, showing that the lateral shift is linearly proportional to the logarithmic scale of the biomolecule concentration.

Finally, these experimental results demonstrate that our proposed prism–SMCW biosensor exhibits an ultra-high sensitivity, which is important for label-free real-time biosensing.

## 5. Conclusions

In this paper, we have presented an ultrasensitive plasmonic biosensor by designing a symmetric metal cladding (SMCW) waveguide structure. We showed that this configuration has significantly improved the detection sensitivity for low-molecular-weight biomolecules due to the giant lateral position of the GH shift associated with a phase singularity. The thickness of the guiding layer was precisely tuned such that the waveguide can accommodate thousands of guided modes, which leads to an extremely large phase change and the subsequent giant lateral position shift from the GH effect with respect to refractive index variations. Here, the GH shift was increased up to 284 µm compared to the one of 18 µm with the four-layered nanophotonic cavity exited at the same wavelength [52]. A series of refractive index detection measurements of glycerol solution at different concentration levels showed a sensitivity figure of merit of 2.33 × 10^4^ µm/RIU, and the lateral shift reaches a value of 90 µm. We have successfully demonstrated the capability of our device in detecting large molecules, such as BSA, as well as small molecules, such as biotin. In summary, the proposed setup has proven its ability to detect ultra-small refractive index (RI) changes (10^−^^8^ RIU), which is of great interest for label-free biosensing application and clinical diagnostics.
